# Influenza virus infection and aerosol shedding kinetics in a controlled human infection model

**DOI:** 10.1128/jvi.01612-24

**Published:** 2024-11-26

**Authors:** Nishit Shetty, Meredith J. Shephard, Nicole C. Rockey, Hollie Macenczak, Jessica Traenkner, Shamika Danzy, Nahara Vargas-Maldonado, Peter J. Arts, Valerie Le Sage, Evan J. Anderson, G. Marshall Lyon, Eric Charles Fitts, Dalia A. Gulick, Aneesh K. Mehta, Mikhael F. El-Chami, Colleen S. Kraft, Krista R. Wigginton, Anice C. Lowen, Linsey C. Marr, Nadine G. Rouphael, Seema S. Lakdawala

**Affiliations:** 1 Department of Civil and Environmental Engineering, Virginia Tech, Blacksburg, Virginia, USA; 2 Department of Civil, Environmental, and Architectural Engineering, University of Kansas, Lawrence, Kansas, USA; 3 Department of Microbiology and Immunology, Emory University School of Medicine, Atlanta, Georgia, USA; 4 Department of Civil and Environmental Engineering, Duke University, Durham, North Carolina, USA; 5 Hope Clinic, Emory University, Decatur, Georgia, USA; 6 Department of Civil and Environmental Engineering, University of Michigan, Ann Arbor, Michigan, USA; 7 Department of Microbiology and Molecular Genetics, University of Pittsburgh School of Medicine, Pittsburgh, Pennsylvania, USA; 8 Department of Medicine, Emory University School of Medicine, Atlanta, Georgia, USA; 9 Department of Pathology and Laboratory Medicine, Emory University School of Medicine, Atlanta, Georgia, USA; St. Jude Children's Research Hospital, Memphis, Tennessee, USA

**Keywords:** influenza, controlled human infection model, antibody response, aerosols, virus shedding, influenza-like illness

## Abstract

Establishing effective mitigation strategies to reduce the spread of influenza virus requires an improved understanding of the mechanisms of transmission. We evaluated the use of a controlled human infection model using an H3N2 seasonal influenza virus to study critical aspects of transmission, including symptom progression and the dynamics of virus shedding. Eight volunteers were challenged with influenza A/Perth/16/2009 (H3N2) virus between July and September 2022 at Emory University Hospital. Viral shedding in the nasopharynx, saliva, stool, urine, and respiratory aerosols was monitored over the quarantine period, and symptoms were tracked until day 15. In addition, environmental swabs were collected from participant rooms to examine fomite contamination, and participant sera were collected to assess seroconversion by hemagglutination inhibition or microneutralization assays. Among the eight participants, influenza virus infection was confirmed in six (75%). Infectious virus or viral RNA was found in multiple physiological compartments, fecal samples, aerosol particles, and on surfaces in the immediate environment. Illness was moderate, with upper respiratory symptoms dominating. In participants with the highest viral loads, antibody titers rose by day 15 post-inoculation, while in participants with low or undetectable viral loads, there was little or no increase in functional antibody titers. These data demonstrate the safety and utility of the human infection model to study features critical to influenza virus transmission dynamics in a controlled manner and will inform the design of future challenge studies focused on modeling and limiting transmission.

**CLINICAL TRIALS:**

This study is registered with ClinicalTrials.gov as NCT05332899.

**IMPORTANCE:**

We use a controlled human infection model to assess respiratory and aerosol shedding kinetics to expand our knowledge of influenza infection dynamics and help inform future studies aimed at understanding human-to-human transmission.

## INTRODUCTION

Influenza virus is a pathogen of global health significance, causing an estimated 290,000–646,000 deaths per year (1). In addition to burdensome seasonal epidemics, influenza viruses cause sporadic pandemics, prompting resource-intensive development of pandemic preparedness plans (2, 3). A major goal of these plans is to limit the spread of infection. Effective measures to reduce transmission require knowledge of the kinetics of viral shedding, the period of contagion, and the efficiency and modes of spread to susceptible individuals.

Controlled human infection model (CHIM) studies have provided an effective approach to track the progression of symptoms alongside viral RNA levels within the nasal cavity in a supervised setting (4–6) and have been employed to test the efficacy of vaccines and antiviral medications for decades (7–11). However, prior CHIM studies have not provided an assessment of the amount of infectious virus found within the respiratory tract, nor have they consistently examined the quantity of influenza viral RNA-containing aerosols expelled from breathing and speaking during a productive infection. This knowledge is critical for understanding influenza virus transmission and mechanisms to prevent forward transmission.

Two human challenge studies have recently been employed to assess human-to-human aerosol transmission of influenza A virus (12, 13). While infectious virus was detected in the respiratory samples of challenged donors in both these studies, the dynamics of shedding from multiple anatomical compartments were not closely examined, and minimal transmission was observed. Additional studies are needed to understand the amount of infectious virus shed in multiple respiratory sites during the course of infection and the expulsion of virus-laden aerosols.

Here, we present the findings of a CHIM study performed at Emory University Hospital, Atlanta, Georgia, from July to September 2022, in which eight participants were intranasally inoculated with seasonal influenza virus (A/Perth/16/2009 [H3N2]). The progression of symptoms and viral shedding in the nasal and oral cavities were monitored daily during the quarantine period. We report infectious viral titers from each specimen type and demonstrate the presence of substantial infectious virus in saliva, a sample type not typically used for the assessment of influenza virus infection. Serum antibody levels were measured both prior to inoculation and periodically for up to 60 days post-inoculation to capture seroconversion. Interestingly, seroconversion was only detected in participants within our high-shedder group (defined as those with cycle threshold [Ct] <25 in nasopharyngeal [NP] swab) and not in low shedders, despite confirmed infection in the low shedders. Finally, levels of viral RNA were examined in participants’ exhaled breath, on environmental surfaces, and in fecal and urine specimens. We observed expulsion of respiratory particles containing viral RNA during breathing and speaking from four participants. Of note, the amount of virus detected in exhaled breath did not correlate with the total number of particles expelled by individual participants. These data provide a useful step toward utilization of CHIM to study the process of influenza virus transmission.

## MATERIALS AND METHODS

### Human study information

A CHIM (ClinicalTrials.gov identifier NCT05332899) was performed at Emory University Hospital from July to September 2022 (Emory IRB protocol STUDY00000083). All participants provided informed consent and passed an assessment of understanding prior to study procedures. Three cohorts, totaling eight individuals, were intranasally inoculated with influenza A/Perth/16/2009 (H3N2) virus, a seasonal strain (manufactured by Meridian Life Sciences, Memphis, TN on behalf of hVIVO, FDA IND #19579). A dose of 5.5 log_10_ median tissue culture dose 50 (TCID_50_/mL) in 0.5 mL was administered to each nostril using an Intranasal Mucosal Atomization Device (MAD Nasal), while the participant was in a supine position. Up to 60 days prior to inoculation, participants were screened for hemagglutinin inhibition (HAI) antibodies against the challenge H3N2 strain. Participants with HAI titers less than or equal to 40 were further screened prior to enrollment. Screening involved medical history, physical examination, laboratory, electrocardiogram (ECG), a negative multiplex upper respiratory pathogen panel (BioFire, Salt Lake City, UT), and chest radiograph imaging. On study day 0, participants were admitted to the hospital and quarantined for 8 days.

### Clinical symptom scores

Symptoms were tracked using an InFLUenza Patient-Reported Outcome (FLU-PRO) diary. Participants completed the FLU-PRO survey every day from study days 1–15. The FLU-PRO survey encompasses questions probing 32 symptoms potentially occurring during an influenza-like illness. Among these 32 symptoms, 11 were selected for analysis here: runny nose, coughing, headache, body ache, tiredness, chills, felt cold, congestion, sore throat, scratchy throat, and sneezing. These specific symptoms were chosen since they are the most prevalent symptoms associated with a typical influenza-like illness, as outlined by the World Health Organization (14). The reported severity of each symptom was assigned a value ranging from 0 (not at all) to 4 (very much). To mitigate bias, scores for runny nose and congestion, scratchy throat and sore throat, and chills and felt cold were averaged for each participant each day and called runny nose/congestion, sore throat, and chills, respectively. This averaging was implemented to address variations in participants’ interpretations of the questions posed regarding these closely related symptoms.

### Specimen collection

**Fig 1:**
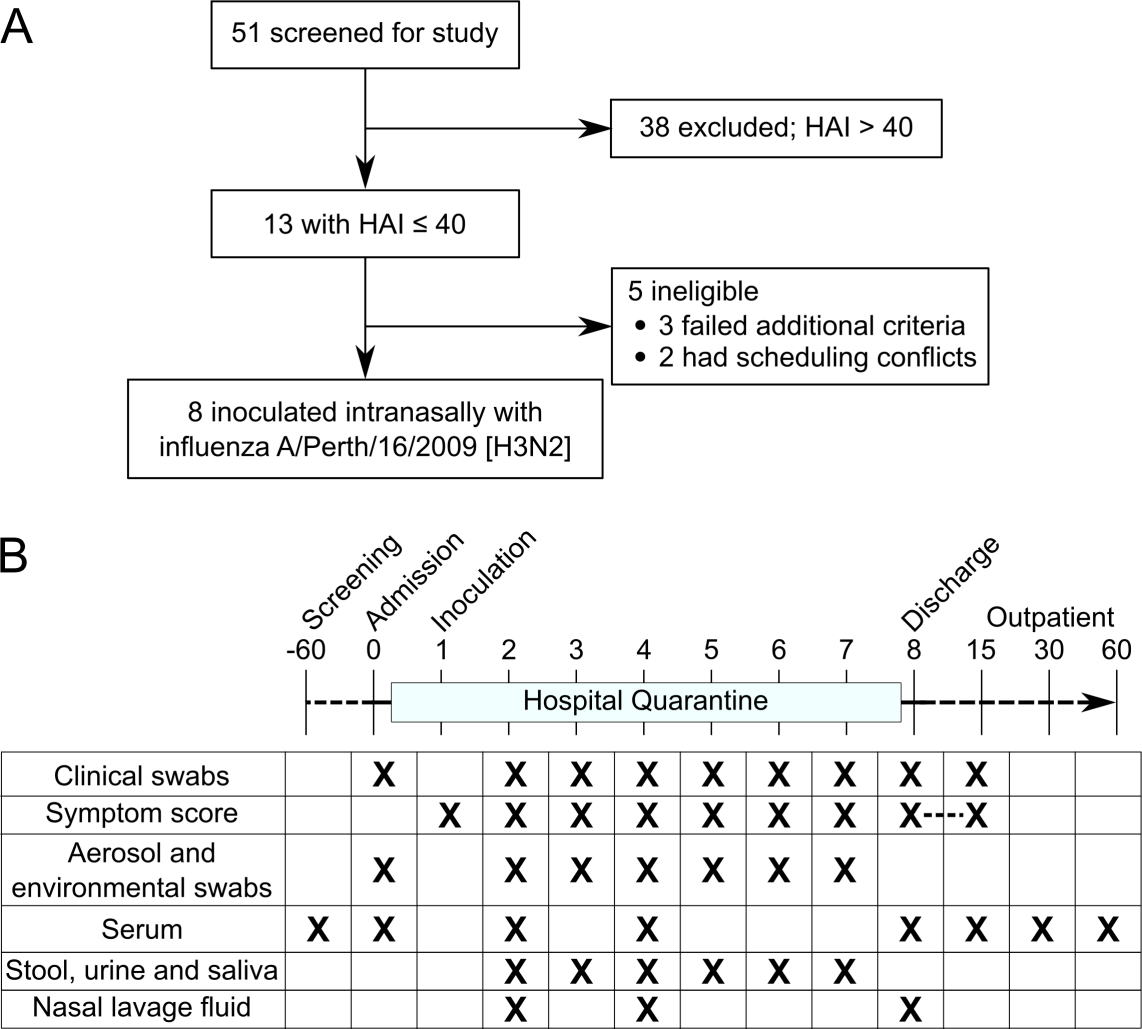
Participant enrollment breakdown and study schedule of events. (**A**) General overview of participant enrollment from recruitment to study conclusion. A total of eight participants were enrolled from July to September 2022. (**B**) Study timeline includes quarantine at the hospital for 7 days post-inoculation. Collection days for different sample types are denoted below the study timeline. Symptom scores from FLU-PRO were collected daily from days 1 to 15. Stool was provided by the participants based on availability. Outpatient visits were conducted on day 15 (±3), 30 (±3), and 60 (±7) post inoculation.

The different sample types and collection days are detailed in Fig. 1B. Further explanation of the methodology is provided below. Respiratory particle emissions from tidal breathing and speaking were sampled for number and presence of virus. Sera were collected to assess seroconversion by HAI and microneutralization (MN) assay. Environmental surface swabs were collected from the bedside control buttons, the overbed tray, the floor underneath the bed, and the bathroom mirror in each participant’s room.

#### Nasopharyngeal sampling methodology

NP sampling was conducted on the day of admission and daily on study days 2–8 (Fig. 1B). Specimens were collected from both nostrils using the same swab unless the swab was saturated with fluid from the first collection. If a deviated septum or blockage created difficulty in obtaining the specimen from one nostril, the same swab was used to obtain the specimen from the other nostril. After collection, the swab was placed tip first into the transport tube (BD Universal Viral Transport Collection Kit, Cat. No. 220526, Fisher Scientific) containing 1 mL of viral transport media. The samples were stored at 2°C–8°C prior to processing for quantitative PCR (qPCR) and subsequently stored in −80°C prior to analysis for infectious virus.

#### Nasal lavage fluid collection methodology

The participant was initially positioned to sit in a semi-recumbent position (45°–70° angle). Sterile tubing was connected to a prefilled syringe for (0.9% NaCl) saline delivery. The tube was primed with saline solution. The participant was asked to place their tongue on roof of their mouth during the procedure to ensure that the soft palate was closed during nasal lavage fluid (NLF) collection. The tubing was inserted into the nostril parallel to the palate. A total of 5 mL of room temperature saline was injected into one nostril and collected in a specimen container held by the participant under their nose. The procedure was repeated for the contralateral nostril for a total of 10 mL total lavage. All NLF was collected in one specimen container and subsequently aliquoted and stored at −80°C prior to analysis. Levels of infectious virus in the NLF were determined by viral plaque assay. Details for this assay are provided in “Virus quantification methods,” below.

#### Saliva collection

Saliva was collected on the day of admission and daily on study days 2–8. The participants did not consume food at least 30 minutes prior to providing saliva. Approximately 3 mL of saliva was collected through spitting into a container (Thermo Fisher, Cat. No. 36319–501). These samples were aliquoted and stored at −80°C prior to analysis. Infectious virus within saliva samples was determined by plaque assay as described in “Virus quantification methods,” below.

#### Stool and urine collection

Participants were provided with stool collection kits (Globe Scientific, Cat. No. 109120L) and urine collection cups (Medical Sales Supply). Stool and urine collection were optional, and samples were provided only by certain participants during the course of the study. These samples were stored at −80°C prior to analysis. Assessment of viral RNA in stool and urine samples was determined by viral RNA quantification as described in “Digital droplet PCR,” below.

#### Serum collection

Serum was collected within 60 days prior to admission for prescreening to assess participant eligibility. Additional serum samples were collected on the day of admission and study days 2, 4, 8, 15 ± 3, 30 ± 3, and 60 ± 7. In this study, we present antibody analysis from sera collected on study days −60, 0, 15 ± 3, 30 ± 3, and 60 ± 7. These sera were processed and analyzed for influenza-specific antibodies with hemagglutinin inhibition and microneutralization assays, details provided in “Serum antibody analysis,” below.

### Aerosol and environmental sampling

#### Respiratory aerosol sampling

Aerosol samples were collected on the day of admission and study days 2–7. The experimental setup for collecting exhaled respiratory particles included a galvanized aluminum funnel with a 20 cm opening. This funnel was connected via a 30 cm length of 3/8” inner diameter conductive silicone tubing to a BioSpot-VIVAS (Aerosol Devices Inc., Ft. Collins, CO) sampler. The sampler gently captures particles through condensation and impaction into a liquid. Particle number concentrations were simultaneously measured using an AeroTrak particle counter (Model 9306-V1, TSI, Shoreview, MN), which detects particles of size 0.3–25 μm. Participants first performed tidal breathing into the funnel for 10 minutes. The collection media were replaced, and the participant subsequently spoke into the funnel for an additional 10 minutes. To measure background aerosol concentrations in the room air, the funnel was subsequently positioned at a distance of approximately 1 m, pointing away from the participant for 10 minutes. BioSpot samples were collected in 1.5 mL phosphate-buffered saline (PBS) with 0.2 M sucrose and 0.5 wt% bovine serum albumin. After collection, samples were placed on ice, aliquoted, and stored at −80°C. Processing for viral RNA levels using digital droplet PCR is described in detail in “Virus quantification methods,” below. Aranet4 HOME monitors were used to measure CO_2_ levels, temperature, and relative humidity (RH) in individual participant rooms where sampling took place. The air change rate in each room was estimated using the CO_2_ decay method (15).

#### Environmental swab collection

Surface swabs were collected from four surfaces in the participant rooms: the bedside control buttons, the overbed tray, the floor underneath the bed, and the bathroom mirror. The bedside control buttons and the overbed tray were swabbed since these were frequently touched surfaces. The area underneath the bed was an untouched surface and any viral RNA found here would be from virus present in environmental aerosols. The bathroom mirror was swabbed to look for virus that may deposit on the surface ballistically from large droplets generated during activities such as teeth brushing. A BD Universal Viral Transport Collection Kit (Fisher Scientific, Cat. No. 220526) was used to collect the environmental swabs. The nylon tip of the provided swab was moistened with transport medium and used to sweep an approximately 10 × 10 cm surface area inside a stencil. The area was swept four times in a crisscross manner while gently rotating the swab to ensure efficient collection. The entire area of the bedside control buttons was swabbed due to their small size. Swabs were placed in the collection tubes containing approximately 1 mL of viral transport media (BD Universal Viral Transport Collection Kit, Fisher Scientific, Cat. No. 220526), vortexed vigorously for 10 seconds, aliquoted, and frozen at −80°C.

#### BioSpot-VIVAS operating parameters and viral RNA gene copies in air calculations

The BioSpot-VIVAS samples air at a flow rate of 8 L/min and collects it onto 1.5 mL of media. The temperature settings of the BioSpot were conditioner = 5°C, initiator = 45°C, moderator = 15°C, and nozzle = 25°C. The sample cooler was not used during the collections.

The concentration of virus RNA measured in gene copies per milliliter using digital droplet PCR (detailed methodology in “Virus quantification methods,” below) was converted to gene copies/L of air using the following equation:


$$Concentration\;of\;virus\;RNA\;in\;air=\;\frac{C_{m}*V_{m}}{Q.t}$$

Where *C*_*m*_ is the concentration of virus RNA in the collection media, *V*_*m*_ is the volume of the collection media (1.5 mL here), *Q* is the air sampling flow rate (8 L/min here), and *t* is the time for collecting samples (10 minutes). The measured RNA concentrations were converted to emission rates by assuming exhaled air flow rates of 6 L/min and 12 L/min for breathing and speaking, respectively.

### Virus quantification methods

A variety of methods were used to quantify the amount of virus within a sample, either by titration of infectious virus or by assessment of the viral RNA through quantitative PCR assays. The description of each method is detailed below.

#### Influenza virus plaque assay

Samples were serially diluted in PBS and added to confluent six-well plates of Madin-Darby Canine Kidney (MDCK) cells. The cells were washed after a 60-minute incubation at 37°C. Minimal Essential Media (MEM) containing 0.7% oxoid agar (Oxoid, United Kingdom), 0.01% diethylaminoethyl-dextran, and 1 µg/mL tosyl phenylalanyl chloromethyl ketone-treated trypsin was added to the cells and allowed to solidify. Cells were incubated for 2 days at 37°C.

#### Viral RNA quantitation

NP swab quantitative PCR assessment: NP swabs collected on the day of admission were used to ensure participant eligibility with a negative multiplex upper respiratory pathogen panel (BioFire, Salt Lake City, UT). Daily NP swabs were tested by PCR-based assay at the Emory University hospital for influenza virus using the Xpert Xpress CoV-2/Flu/RSV plus test (Cepheid, Sunnyvale, CA); this includes two distinct primer sets to the influenza genome. PCR test results were provided in Epic, and Ct values were obtained from the clinical lab director.

#### Viral RNA extraction

Nucleic acids were extracted from environmental swab samples using the MagMAX Viral/Pathogen nucleic acid isolation kit (Thermo Fisher, Cat. No. A42352) on the KingFisher Duo Prime (Thermo Fisher) purification system. Respiratory samples were extracted using the QIAamp Viral RNA Mini kit (Qiagen, Cat. No. 52904). Stool sample processing and extraction protocols were adapted from previously published procedures for SARS-CoV-2 RNA analysis in fecal samples (16). Total nucleic acid from stool was extracted with the Chemagic Viral DNA/RNA 300 kit H96 (Perkin Elmer, Cat. No. CMG-1033-S) using the Chemagic 360 automated extraction platform (Perkin Elmer, Cat. No. CMG-360).

#### Quantitative PCR for viral RNA in environmental samples

Influenza virus RNA from the surface swab sample extractions was measured by qPCR using the iTaq Universal Probes One-Step kit (Bio-Rad, Cat. No. 1725141) with 450 nM of primers and 250 nM of probes targeting the M gene segment. Primer/probe sequences are forward primer 5′-AGATGAGTCTTCTAACCGAGGTCG-3′, reverse primer 5′-GCAAAGACATCTTCAAGTCTCTG-3′, and probe 5′-FAM-TCAGGCCCC/ZEN/CTCAAAGCCGA-BHQ1-3’. The reaction was run on a CFX Connect real-time PCR system (Bio-Rad) using the following cycling conditions: 50°C for 5 minutes, 95°C for 1 minute, 40 cycles of 95°C for 10 seconds, and 60°C for 20 seconds.

#### Digital droplet PCR

Stool samples were stored at −80°C prior to analysis and thawed on ice prior to processing. Subsamples of each stool specimen were precisely weighed and suspended in 1,200 µL of DNA/RNA shield (Zymo, Cat. No. R1100). Concurrently, suspensions were homogenized for 2 minutes using a Biospec bead beater (Biospec, Cat. No. 1001) and 0.5 g silica zirconia beads (Biospec, Cat. No. 11079105z) per tube. Total nucleic acid was extracted in duplicate from the homogenates using the Chemagic 360 automated extraction instrument and the Chemagic Viral 300 kit (Revvity, Cat. No. CMG-1033-S). Urine samples were processed with the Chemagic directly, using duplicate 300 µL samples.

Concurrent to nucleic acid extraction, the dry mass fraction of each stool sample was measured. A precisely measured subsample of stool (approximately 1 g) was added to a weighed microcentrifuge tube with a hole in the lid for ventilation. Tubes were heated at 100°C for at least 8 hours in a biosafety cabinet, and the samples were weighed again. The dry mass fraction was calculated as follows:


$$f_{dry\;mass}=\frac{mass\;of\;dried\;sample-mass\;of\;tube}{mass\;of\;fresh\;sample-mass\;of\;tube}$$

ddPCR was used to measure influenza viral M gene segment in respiratory samples, stool, and urine extracts using the One-step RT-ddPCR Advanced kit for Probes (Bio-Rad, Hercules, CA). Reactions for testing respiratory sample extracts used the primers and probe listed above with each primer at 1 µM and the probe at 250 nM. Reactions for testing urine and stool sample extracts contained 450 nM forward primers 5′-CAAGACCAATCYTGTCACCTCTGAC-3′ and 5′-CAAGACCAATYCTGTCACCTYTGAC-3′, 675 nM reverse primer 5′-GCATTYTGGACAAAVCGTCTACG-3′, 225 nM reverse primer 5′-GCATTTTGGATAAAGCGTCTACG-3′, and 250 nM probe 5’-/ATTO590/TGCAGTCCTCGCTCACTGGGCACG/3IABRQSp/−3’ (17). Thermal cycling procedure was as follows: 50°C for 1 hour, 95°C for 10 minutes, 40× (94°C 30 s, 56°C 1 min), 98°C 10 minutes, and 4°C hold. The ddPCR reactions for stool and urine were processed and analyzed using a QX600 AutoDG Droplet Digital PCR System (Bio-Rad).

Duplicate sample extractions were analyzed as single reactions on 96-well plates. In addition to samples, each PCR plate included duplicate negative extraction controls (nuclease-free water processed as sample through extraction steps), PCR no template controls (nuclease-free water added to PCR reactions prior to thermal cycling), and gBlock synthetic DNA positive controls Integrated DNA Technologies (IDT). All ddPCR data were thresholded in QX Manager v2.1 using the positive controls as a reference for expected amplitude. Limits of blank (LOB) for each ddPCR plate were set as the total upper 95% confidence limit calculated for the negative extraction controls, as calculated by the QX Manager software. Further analysis was performed in R (R studio, v4.1.2). Analytical limits of detection (LOD) were calculated from the LOB using the following equation:


LOD=mean(LOB)+1.96∗sd(LOB)

Measurements were converted to a physiologically relevant basis (copies/mg-dw for stool, copies/mL for urine), using dry mass measurements, extracted masses and volumes, and PCR template and reaction volumes.

### Serum antibody analysis

Antibody assessment was used to determine participant eligibility through a hemagglutination inhibition (HAI) titer of ≤40. Detection of functional antibodies post challenge was evaluated for both HAI and MN assays, both detailed below.

#### Serum treatment

Prior to performing HAI and MN assays, one-part sera was incubated with three parts receptor destroying enzyme (RDE; Hardy Diagnostics, Lot No. 682101) overnight at 37°C and then heat inactivated at 56°C for 30 minutes.

#### Viruses used in HAI and MN assays

The A/Perth/16/2009 (H3N2) virus used in HAI and MN assays was generated from subsequent egg passage of the residual inoculum. The H1N1 virus used for HAI and MN assays was generated by reverse genetics plasmids for A/California/07/2009 (H1N1), gifts from Dr. Jesse Bloom (Fred Hutch Cancer Research Center, Seattle). Once rescued, the H1N1 virus was passaged in MDCK cells. The H3N2 virus was treated as homologous to the hVivo challenge virus (A/Perth/16/2009), and the H1N1 virus is a complementary seasonal H1N1 virus. Titers for both viruses were determined by defining the tissue culture infectious dose 50.

#### HAI assay

For the HAI assay, six parts of normal saline was added for a final concentration of 1:10 sera. Serial twofold dilutions of RDE-treated sera were prepared in a V-bottom microtiter plate starting with a 1:10 dilution. Dilutions of H3N2 and seasonal H1N1 viruses were created to achieve HA titer of 8 HAU/50 µL. Diluted virus was added and incubated at room temperature for 15 minutes. Turkey red blood cells (tRBC; Lampire, Lot No. 23K28041) were added at a concentration of 0.75% and incubated for 40 minutes at room temperature. HAI titers were determined by the reciprocal of the highest dilution of sera that inhibited hemagglutination. Hemagglutination inhibition was defined as the prevention of clumping of tRBCs indicated by the presence of a teardrop shape forming at the bottom of the V-bottom well as the plate was rotated vertically.

#### MN assay

For analysis of neutralizing antibody titers by MN assay, six parts normal saline were added for a final concentration of 1:10 RDE-treated sera. Serial twofold dilutions were prepared in 1× MEM starting at 1:10 dilution of RDE-treated sera. Equal volumes of sera and homologous H3N2 virus and seasonal H1N1 virus at a concentration of 3.3 log_10_ TCID_50_/mL were mixed and incubated at room temperature for 60 minutes. Virus dilutions were made in MEM containing 0.5% bovine serum albumin (Sigma-Aldrich, Cat. No. A3294). Residual infectivity of the serum-virus mixture was assessed following incubation using MDCK cells seeded to confluency 1–3 days prior in 96-well microtiter plates. The concentration of neutralizing antibodies was calculated by taking the reciprocal of the highest dilution of serum needed to completely neutralize 2 log_10_ TCID_50_ of the virus. Neutralization was defined as the absence of cytopathic effect on day 4 post incubation of the virus-antibody mixture.

## RESULTS

### Study participants

Fifty-one volunteers between the ages of 18 and 49 were screened for serological susceptibility to the challenge strain (influenza A/Perth/16/2009 [H3N2] virus) by HAI assay (Fig. 1A). Thirteen volunteers with HAI titers less than or equal to 40 were further screened for additional criteria, and eventually, eight participants were enrolled and admitted into single occupancy rooms 1 day prior to administration of the virus (study day 0). On study day 1, the participants were inoculated intranasally with influenza A/Perth/16/2009 (H3N2) virus. The study schedule of events is detailed in Fig. 1B. Participants without two consecutive negative qPCR tests at discharge were given oseltamivir phosphate.

### Virus shedding dynamics varied by individual

**Fig 2:**
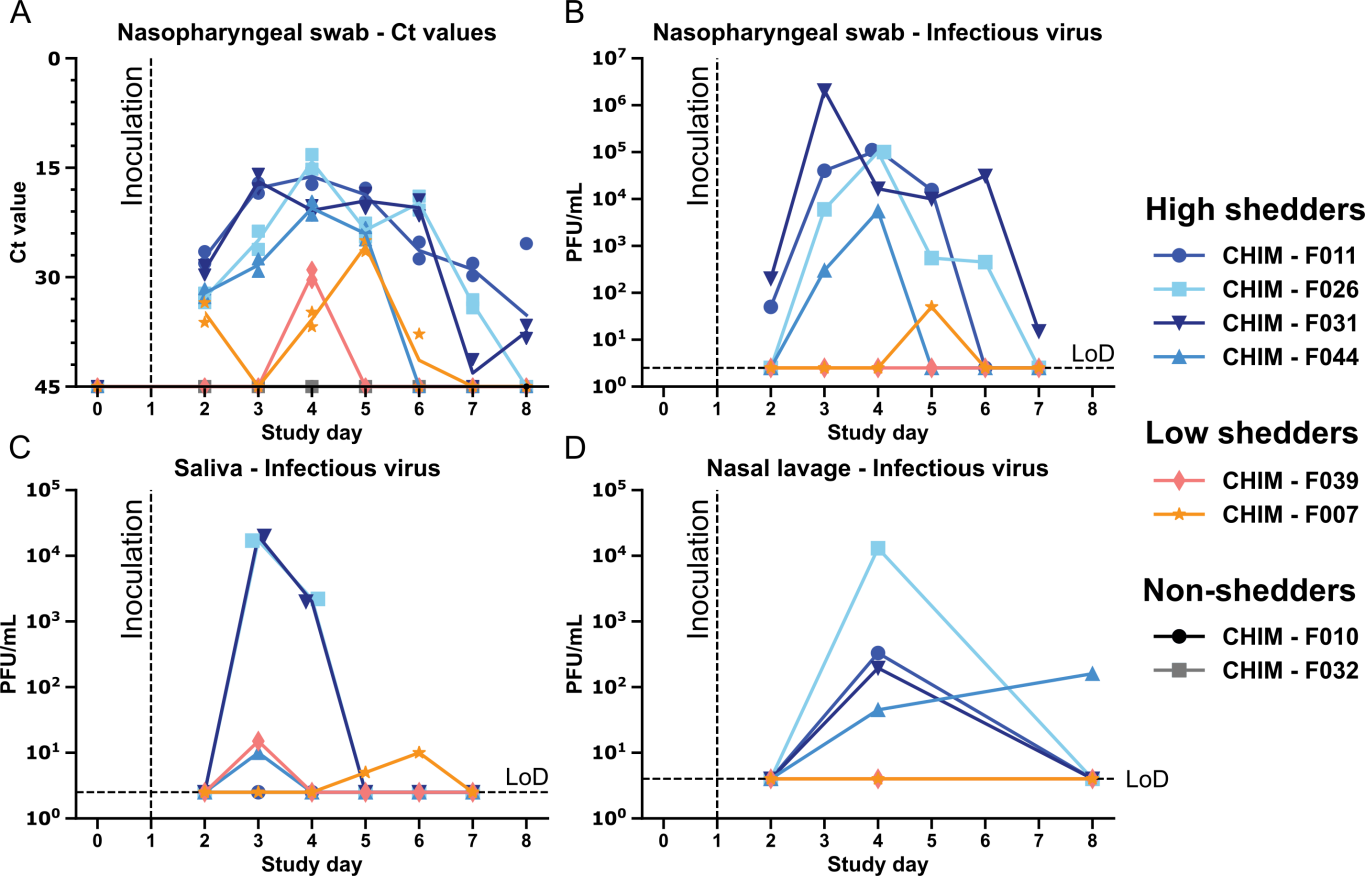
Viral shedding in nasal and oral cavities. (**A**) qPCR Ct values for NP swabs. Each sample was run with two distinct influenza A virus primers (see Materials and Methods for details). Amount of infectious virus present in (**B**) NP swabs, (**C**) saliva, and (**D**) NLF was determined from participants who tested PCR positive. The dashed horizontal line is the LOD for plaque assay, and the dashed vertical line represents the day the participants were inoculated.

**Fig 3:**
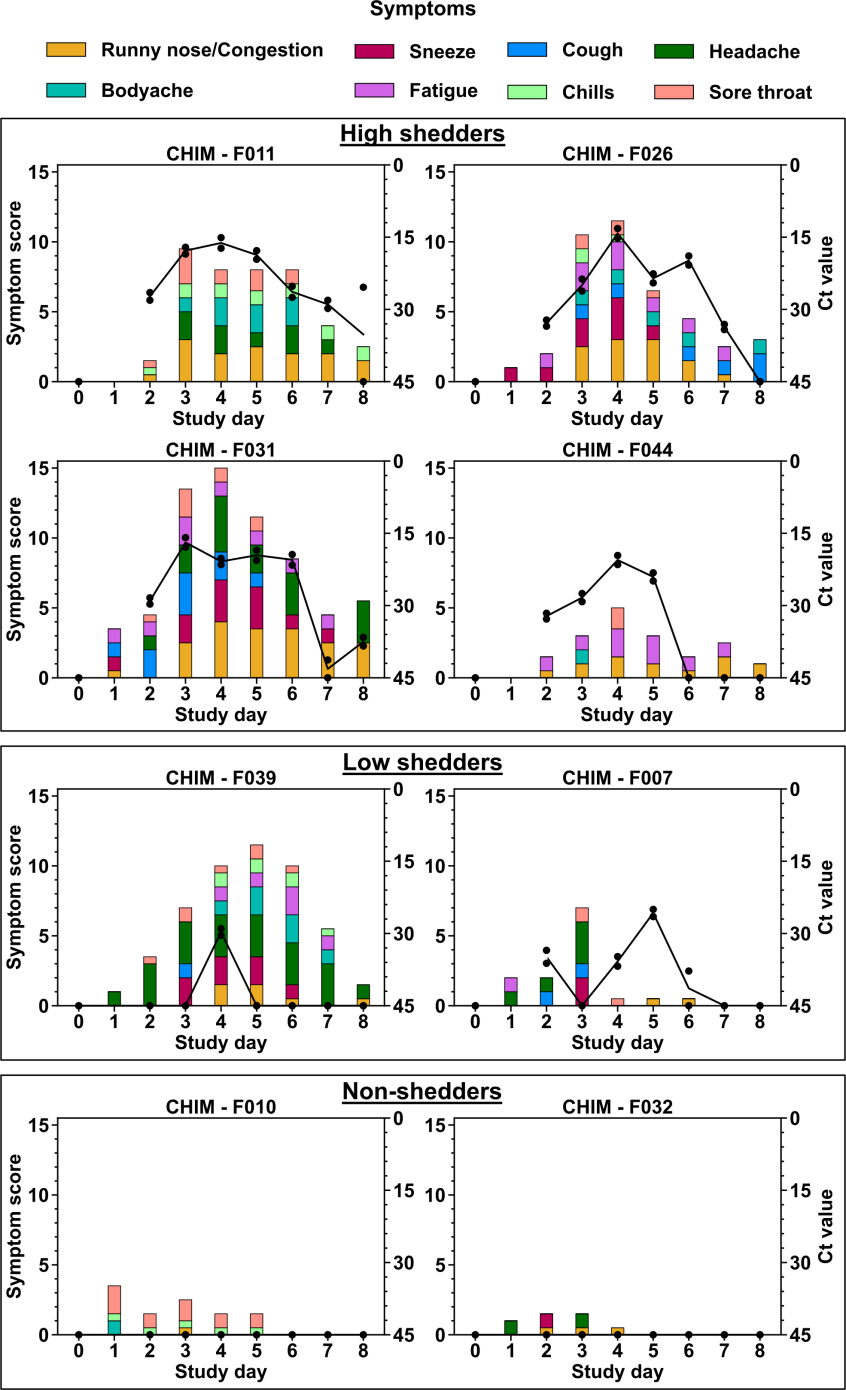
Daily participant symptom scores and NP viral RNA shedding. The columns show symptom scores for each participant with each color indicating a specific symptom. The solid lines represent Ct values for qPCR from the NP swabs with the symbols representing two separate gene targets.

Over the quarantine period, daily NP swabs were collected and tested for influenza virus by qPCR. Six of the eight participants were PCR positive on at least 1 day during the course of the study and displayed symptoms consistent with influenza infection (Fig. 2A and 3), achieving an attack rate of 75% based on mild-to-moderate influenza disease as previously defined by (5). Four participants (50%) had multiple days of PCR positivity including at least one sample with Ct values <25; these individuals are herein referred to as “high shedders.” Two participants (25%) had PCR positivity on at least 1 day with peak qPCR Ct values ≥25 (herein referred to as “low shedders”), and two participants (25%) remained PCR negative for the duration of the study (herein referred to as “non-shedders”; Table 1; Fig. 2A).

**TABLE 1:** Participant information and summary of results

CHIM identifier	High shedders^*h*^				Low shedders^*h*^		Non-shedders^*h*^	
	F011	F026	F031	F044	F007	F039	F010	F032
Sex	Female	Female	Female	Female	Male	Female	Female	Female
Age	45	36	28	28	35	35	36	20
Race	Black	White	White	Black	Asian	Black	White	White
Ethnicity	Non-Hispanic	Non-Hispanic	Hispanic	Non-Hispanic	Non-Hispanic	Non-Hispanic	Non-Hispanic	Non-Hispanic
BMI^*a*^	24.2	20.9	34.2	29.5	28	30.7	28.8	34.2
Maximum symptom score (study day)^*b*^	35 (3)	23 (4)	32 (4)	20 (4)	8 (3)	33 (5)	4 (3, 4)	3 (3)
HAI titer								
Screen^*c*^	10	10	20	10	10	10	20	10
Fold raise^*d*^	5.71	5.71	4.07	18.14	1.43	1.79	1.60	1
Infectious virus^*e*^								
NP swab	++	++	++	++	+	−	NA^*g*^	NA
Saliva	−	++	++	+	+	+	NA	NA
NLF	+	++	+	+	−	−	NA	NA
Viral RNA^*f*^								
Stool	−	+	+	−	−	−	−	−
Urine	NA	−	−	−	−	−	NA	−
Breathing (aerosol)	+	−	−	+	−	−	NA	NA
Speaking (aerosol)	−	−	+	+	+	−	NA	NA
Fomites	+	+	−	−	−	−	NA	NA

^*a*^ BMI = body mass index in kg/m^2^.

^*b*^ Symptom scores calculated from FLU-PRO survey. Maximum cumulative score for all 32 symptom questions is presented, and the day corresponding to this peak in symptoms is indicated in parentheses.

^*c*^ HAI titers against A/Perth/16/2009 (H3N2).

^*d*^ Fold raise = highest geometric mean fold change in the antibody titers post-inoculation.

^*e*^ “++” denotes peak infectious virus titer >10^3^ pfu/mL, “+” denotes peak infectious titer ≤10^3^ pfu/mL, and “−” denotes no infectious virus detected through plaque assay.

^*f*^ “+” denotes at least one positive sample, and “−” denotes no positive sample.

^*g*^ NA = sample not analyzed.

^*h*^ High shedders = participants with peak NP swab Ct value <25; low shedders = participants with peak NP swab Ct value ≥25; Non-shedders = participants without a PCR positive on any study day.

Peak levels of viral RNA from NP swabs were observed on study days 3–5 (Fig. 2A). Infectious virus titers from NP swabs and NLF generally followed the same trend for high-shedding participants (Fig. 2B and D). Only one low shedder (F007) had detectable infectious virus from NP swabs but not the NLF (Fig. 2B and D). The shedding of infectious virus in saliva was more sporadic compared to that seen in the nasal site (Fig. 2C). Although high shedders F026 and F031 had substantial infectious titers in their saliva on days 3 and 4, similar to what was observed in NP swabs, high shedder F044 exhibited infectious virus in saliva only on day 3 at a lower titer, while high shedder F011 did not have detectable infectious virus. Surprisingly, both the low shedders (F007 and F039) had infectious virus in their saliva, suggesting that sampling of a single site for detection of influenza virus may not capture the full spectrum of the viral shedding during infection.

Detection of viral RNA in wastewater is proving to be an important strategy in surveillance of respiratory viruses such as SARS-CoV-2 and influenza (18–20). To determine the connection between respiratory/oral viral shedding and detection of viral RNA in excrements, stool and urine samples from the infected participants, when available, were assessed for viral RNA. High shedders F026 and F031 had detectable viral RNA in stool on study days 6 and 7, with genome copies per milligram of dry weight being 380 and 9,521, respectively (Table 1). These 2 days correspond with the detection of infectious virus in the NP swabs of these participants but not in the other sites collected. Detailed shedding kinetics in stool could not be established due to a lack of sample availability. None of the urine samples had viral RNA above the limit of detection. Detection of viral RNA in 33% (2/6) of the fecal samples toward the end of the quarantine period is consistent with previous studies (21, 22). The two participants with viral RNA-positive stool samples (F026 and F031) also exhibited higher infectious virus titers in saliva a few days prior, suggesting that viruses in the oral cavity enter the digestive system when swallowed. However, not all participants provided a stool sample daily, making it challenging to establish shedding kinetics. Future studies with more participants will be required to determine when viral RNA is expected to be excreted into wastewater during infection.

### Disease presentation corresponds with viral load

Clinical symptoms were monitored and scored daily using a FLU-PRO survey (23) (Fig. 3). The most commonly experienced symptoms were runny nose and nasal congestion (100%), followed by headache (67%). PCR positivity in the NP swabs occurred 1 day prior to onset of clinical symptoms for the high shedders. However, peak presentation of symptoms coincided with peak viral load measured by qPCR in NP swabs (Fig. 3). On average, the highest symptom scores and peak NP viral loads were observed on study day 4 (3 days post inoculation; Table 1). Generally, the high shedders presented with more and persistent clinical symptoms compared to low and non-shedders. Interestingly, for F039 (low shedder), there was a lack of consistent viral shedding, yet this participant reported a wide range of symptoms.

### Aerosol shedding

**Fig 4:**
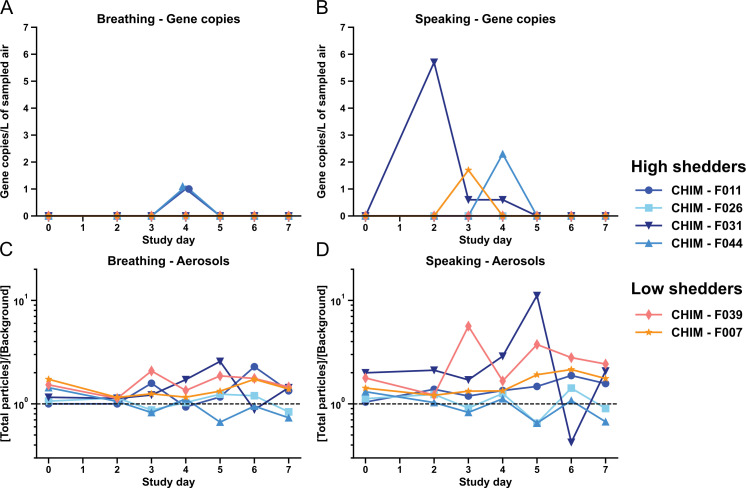
Viral RNA and aerosol particles in respiratory emissions. The number of viral gene copies in aerosol expulsions was determined using droplet digital PCR during (**A**) breathing and (**B**) speaking. Aerosol particle concentrations measured during (**C**) breathing and (**D**) speaking. Measured particle concentrations (for particles of 0.3–25 µm size range) were normalized to background particle concentrations in the corresponding participant’s room. The dashed horizontal line indicates a ratio of 1 with respect to the background.

Respiratory aerosol expulsions of infected participants were evaluated for the presence of viral RNA during breathing and speaking (Fig. 4A and B). We observed that the average amount of viral RNA expelled during speaking was greater than that measured during breathing. Viral RNA was detected in breathing emissions for two high-shedding participants (F011 and F044) and speaking emissions for three participants (high shedders F031 and F044 and low shedder F007). Only high shedder F031 expelled virus during speaking that could be detected across multiple days. This participant also had a higher level of infectious virus measured in their saliva. No infectious virus was detected in any aerosol samples, perhaps due to low amounts of virus expelled in respiratory emissions and/or inactivation during collection and freeze-thaw cycles.

**Fig 5:**
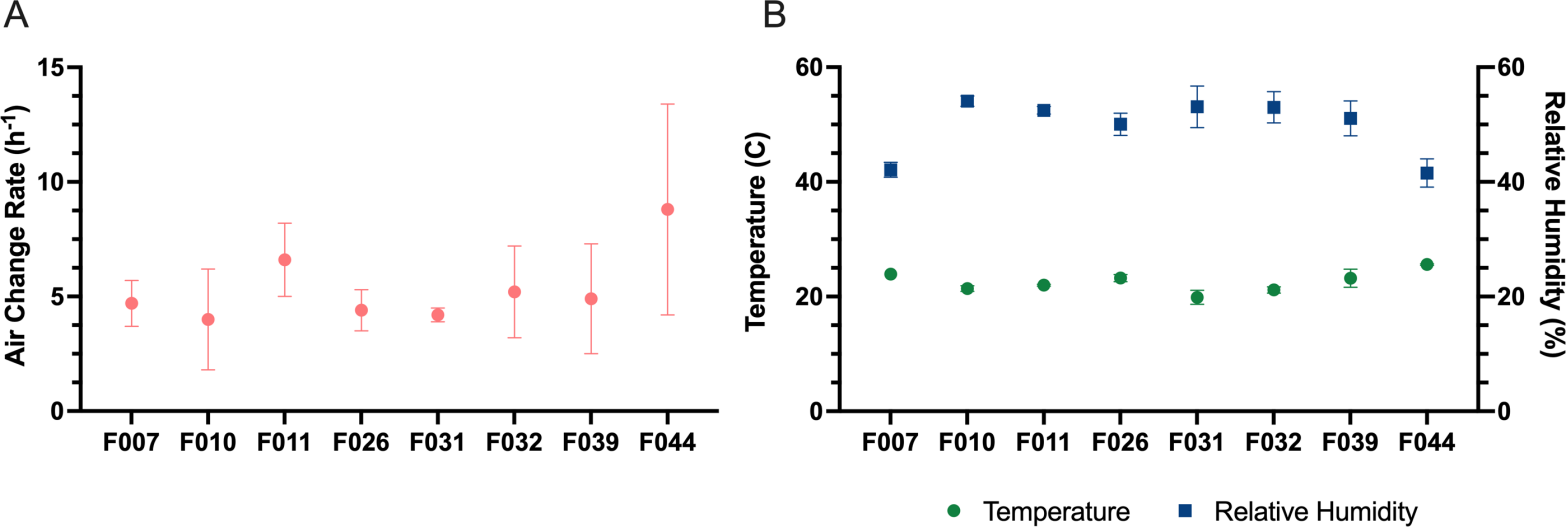
Environmental conditions in participant rooms. (**A**) Average air change rates in individual participant rooms during the course of their hospital stay. (**B**) Daily average temperature and RH in participants’ rooms, measured next to the bed, during the course of their hospital stay. Error bars represent the SD across study days 2–7.

To determine whether the amount of viral RNA expelled was correlated with the number of total respiratory particles expelled, we measured total particle concentrations during breathing and speaking (Fig. 4C and D). The measurements represent a mixture of particles in participants’ exhaled breath and in background room air. To account for differences in background, total particle concentrations were normalized to the background levels in each participant’s room. Average particle concentrations expelled during speaking were greater than average particle concentrations during breathing, although this difference was not statistically significant (*P* = 0.14) due to large variations in emissions during the sampling period. Background levels of particles varied by room due to differences in air change rates and variability in particle emission rates from all sources within the room. Average air change rates ranged from 4 to 9 air changes per hour (Fig. 5A). Average temperature ranged from 19.9°C to 25.6°C, and average RH ranged from 42% to 54% (Fig. 5B). Although high shedder F031 exhibited both the highest concentration of viral RNA and highest average particle number emissions relative to background, we did not observe a strong association between detected viral gene copies and expelled particle concentrations (*R*^2^ = 0.02). Overall, the amount of virus found in respiratory particles was not correlated with the number of particles expelled in this study, although this requires further investigation.

### Environmental sampling

Given the presence of viral RNA-laden aerosols expelled by many of the participants, we explored whether there was detectable influenza viral RNA on surfaces within the hospital rooms as well. Four sites (floor under the bed, mirror, tray, and bed buttons) were sampled every day from study days 2–7 and then processed for viral RNA extraction. Viral RNA was only detected on frequently touched surfaces from the rooms of two high shedders: the overbed tray of F011 on days 4 (Ct = 33.9), 5 (Ct = 28.9), and 6 (Ct = 33.0) and the bedside control buttons of F026 on days 4 (Ct = 37.4) and 5 (Ct = 35.4). Infectious viral shedding was detectable for both participants on days 4 and 5. Viral RNA-containing aerosols were detected in F011 breath on day 4 but not on any day for F026. All other environmental surface swabs were negative for viral RNA, and the surface swabs were not assessed for infectious virus. Room ventilation rates and environmental factors could have impacted the ability to detect viral RNA on surfaces within the room environment.

### Seroconversion corresponds to high viral shedding

**Fig 6:**
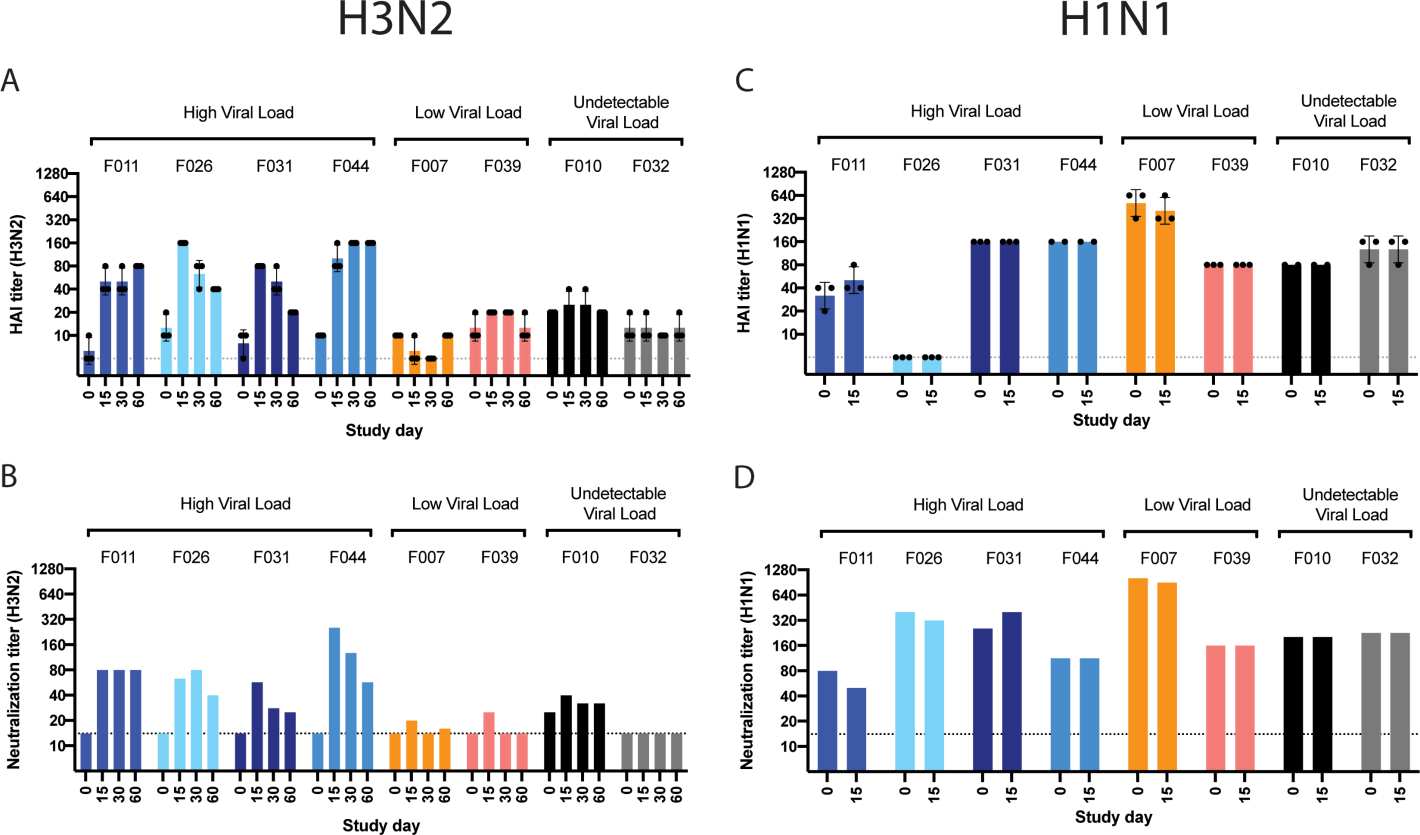
Serological responses to A/Perth/16/2009 (H3N2) and A/California/07/2009 (H1N1) virus. Sera from the study participants were tested for antibodies capable of (A and C) inhibiting hemagglutination of red blood cells or (B and D) neutralizing infection of cultured cells against A/Perth/16/2009 (H3N2) or A/California/07/2009 (H1N1), respectively. The HAI assays were run in triplicates, and MN assays were run as one replicate. The dashed line shows the limit of detection for each assay. The geometric mean for the three HAI replicates was plotted, and the SD is shown.

A fourfold rise in HAI antibody titer after infection is the conventional threshold for seroconversion. All four participants with high viral shedding had a fourfold rise in HA-specific antibodies to A/Perth/16/2009 (H3N2) virus, while both participants with undetectable viral loads exhibited no evidence of seroconversion by HAI assay (Fig. 6A). Interestingly, neither of the two participants with low viral loads exhibited a fourfold rise in HAI titers despite shedding infectious virus from nasal and oral cavities. MN data revealed similar results; neutralizing antibody titers against the challenge virus confirmed seroconversion in all four participants with high viral loads (Fig. 6B). Participants with low or undetectable viral loads, however, had no evidence of an increase in neutralizing antibody levels.

Infection with influenza viruses can induce the production of antibodies generated during an individual’s first influenza infection, in a process known as original antigenic sin. To assess a rise in antibody responses to circulating seasonal influenza viruses among participants, the presence of functional antibodies against A/California/07/2009 (H1N1pdm09) virus was determined. None of the eight participants, regardless of respiratory viral shedding kinetics, displayed a rise in HA-specific antibodies against H1N1pdm09 virus (Fig. 6C). Similarly, no significant change in neutralizing antibody titers against the H1N1pdm09 virus was observed in any of the study participants (Fig. 6D). Overall, robust functional antibody responses were generated primarily in high-shedding participants and were specific to the H3N2 challenge virus.

## DISCUSSION

We report a successful deployment of a CHIM to evaluate influenza viral load in different respiratory compartments and detection of viral RNA in aerosols released during speaking and breathing as well as on surfaces in the vicinity of experimentally infected individuals. Our observed attack rate for challenged participants was 75%, in line with recent H3N2 challenge studies that reported attack rates of 78% and 81% at similar doses (12, 13). Peak symptom scores were observed on study day 4, also similar to other CHIM studies (24–26). The CHIM described is, however, distinct from other contemporary CHIM in that (i) the H3N2 strain used in these studies differs from those in references (5, 12, 24, 25); (ii) we report the presence of infectious virus in the oral cavity, which is not a commonly sampled site; and (iii) we assessed the timing and amount of viral RNA expelled into the environment. These results shed light on the transmission potential of mild-to-moderate influenza infections.

The use of CHIM studies to assess the expulsion of influenza virus into the air is an important aspect of using this model system to explore influenza virus transmission. In our study, viral RNA was detected from aerosols for 67% (4/6) of participants, but only F031—the participant with the highest particle expulsions—had detectable viral RNA across multiple days. We did not observe a strong association between expelled viral RNA and symptom score (or reported sneezing). There was also no association between expelled viral RNA and total aerosol particle concentration, despite the aerosol measurements of F031. However, these relationships will need to be examined again in future studies since the high concentration of particles in background air, low number of positive samples, and low number of participants is likely to have obscured any meaningful correlations in this study. Our results equated to an emission rate ~6–70 gene copies per minute, which is consistent with previous measurements of influenza virus in exhaled breath (27, 28).

Detection of infectious influenza viruses from the air is challenging since it requires (i) sampling systems that retain the infectivity of the virus and (ii) the collection of a sufficient amount of air to detect low levels of virus expelled into the environment (29, 30). Given these challenges, it is not surprising that infectious virus was not detectable from respiratory emissions in our studies. Previous estimates suggest that there is at least a 10- to 100-fold difference between infectious virus and viral RNA in exhaled breath (27), thus the amount of virus-laden aerosols released by the participants may have been too low to be sufficiently collected in the 10-minute collection time. Other published attempts at collecting infectious influenza virus from exhaled breath aerosols have also yielded low detection rates and/or low amounts of infectious virus (31–35). The low detection rates of viral RNA-containing aerosols in our study may be related to shorter collection times than previous studies (32, 33). The collection efficiency of the BioSpot-VIVAS model to capture particles larger than ~8 µm is limited, resulting from losses at the inlet, and could have also contributed to the low detection rate. Aerosol sampling platforms that capture larger aerosols and retain pathogen infectivity are needed to better quantify viral RNA and infectious virus from respiratory emissions. This is an active area of further investigation by our group.

While the four participants with the highest levels of virus shedding had increases in HAI and MN titers, indicating that humoral immune responses were activated in response to infection, two infected participants with low viral loads did not have these increases in titers that would have been expected with seroconversion. This is similar to previous studies which also observed no increase in HAI titers for some participants after exposure to influenza virus (4, 36). These observations suggest that the level or anatomical location of viral replication may be determinants of the humoral immune response and whether antibody seroconversion occurs. While our numbers are relatively small, our data suggest assessing seroconversion using HAI or MN assays on serum may be insensitive to infections that result in low-level virus replication. Additional studies examining mucosal immunity, specifically antibodies in the upper respiratory tract, may better reflect infection compared to systemic antibody responses.

Although our pilot study provides valuable insights, it highlights multiple areas that need further refinement prior to the establishment of a human-to-human transmission model. First, future air sampling can be improved by isolating respiratory emissions from background particles and eliminating variations introduced from conducting sampling in rooms with different ventilation rates. Second, our sampling setup was designed to primarily sample virus in aerosol particles <25 µm in size and missed virus in larger particles. A sampling platform capable of detecting respiratory particle emissions encompassing a wide range of sizes, which include larger droplets from coughing and sneezing, is necessary to comprehensively characterize viral shedding. Finally, CHIM studies have relied on intranasal instillation to inoculate participants, which does not replicate the natural infection route and may not fully represent the clinical disease or viral expulsions observed in naturally infected individuals. In contrast, aerosol-based inoculation methods more closely mimic natural infection and offer a more effective approach for infecting participants. While aerosol inoculation has been abandoned for decades due to safety concerns (37), advancements in aerosol generation technologies and the protective effects of prior immunity could provide a pathway to reestablish this method of controlled infection and should be reexamined for use in future studies.

Evaluating interventions to mitigate influenza virus transmission requires understanding of a large number of contributing factors, including viral phenotypes, environmental conditions, transmission kinetics, and host susceptibility. CHIM provides a valuable approach for investigating these features in a supervised environment. Our CHIM study strengthens existing understanding of viral shedding during symptomatic influenza virus infection and expands knowledge of the dynamics of infection and shedding, which will inform the design of CHIM transmission studies.

## Data Availability

All the data on viral titers, particle sizes, and antibody data used to generate the graphs presented in this manuscript are available on FigShare at DOI: https://doi.org/10.6084/m9.figshare.25954900. Also available are the complete FLU-PRO questionnaires for all the enrolled participants.
