# Correction for Shetty et al., “Influenza virus infection and aerosol shedding kinetics in a controlled human infection model”

**DOI:** 10.1128/jvi.01354-25

**Published:** 2025-10-07

**Authors:** Nishit Shetty, Meredith J. Shephard, Nicole C. Rockey, Hollie Macenczak, Jessica Traenkner, Shamika Danzy, Nahara Vargas-Maldonado, Peter J. Arts, Valerie Le Sage, Evan J. Anderson, G. Marshall Lyon, Eric Charles Fitts, Dalia A. Gulick, Aneesh K. Mehta, Mikhael F. El-Chami, Colleen S. Kraft, Andrew P. Catchpole, Alex Mann, Krista R. Wigginton, Anice C. Lowen, Linsey C. Marr, Nadine G. Rouphael, Seema S. Lakdawala

## AUTHOR CORRECTION

Volume 98, no. 12, e01612-24, 2024, https://doi.org/10.1128/jvi.01612-24. The byline should appear as given in this correction. Andrew P. Catchpole and Alex Mann were inadvertently omitted.

Page 16: Affiliation 10 should appear as follows, for added authors Andrew P. Catchpole and Alex Mann: “hVIVO, London, United Kingdom.” 

